# Mental Health Status of Sri Lanka Navy Personnel Three Years after End of Combat Operations: A Follow Up Study

**DOI:** 10.1371/journal.pone.0108113

**Published:** 2014-09-25

**Authors:** Raveen Hanwella, Nicholas E. L. W. Jayasekera, Varuni A. de Silva

**Affiliations:** 1 Department of Psychological Medicine, Faculty of Medicine, University of Colombo, Colombo, Sri Lanka; 2 General Health Services, Sri Lanka Navy, Colombo, Sri Lanka; Univ of Toledo, United States of America

## Abstract

The main aim of this study was to assess the mental health status of the Navy Special Forces and regular forces three and a half years after the end of combat operations in mid 2009, and compare it with the findings in 2009. This cross sectional study was carried out in the Sri Lanka Navy (SLN), three and a half years after the end of combat operations. Representative samples of SLN Special Forces and regular forces deployed in combat areas were selected using simple random sampling. Only personnel who had served continuously in combat areas during the one year period prior to the end of combat operations were included in the study. The sample consisted of 220 Special Forces and 275 regular forces personnel. Compared to regular forces a significantly higher number of Special Forces personnel had experienced potentially traumatic events. Compared to the period immediately after end of combat operations, in the Special Forces, prevalence of psychological distress and fatigue showed a marginal increase while hazardous drinking and multiple physical symptoms showed a marginal decrease. In the regular forces, the prevalence of psychological distress, fatigue and multiple somatic symptoms declined and prevalence of hazardous drinking increased from 16.5% to 25.7%. During the same period prevalence of smoking doubled in both Special Forces and regular forces. Prevalence of PTSD reduced from 1.9% in Special Forces to 0.9% and in the regular forces from 2.07% to 1.1%. Three and a half years after the end of combat operations mental health problems have declined among SLN regular forces while there was no significant change among Special Forces. Hazardous drinking among regular forces and smoking among both Special Forces and regular forces have increased.

## Introduction

Several studies have shown that, in military personnel, exposure to combat has adverse mental health consequences. Post-traumatic stress disorder (PTSD) was first described in Vietnam war veterans. PTSD is not the only adverse effect of combat exposure. Exposure to combat is also associated with increased risk of depression, alcohol misuse and multiple physical symptoms [1]–[4]. These can result in impairment of occupational and social functioning and increased use of health care services [3]. In recent times, studies of United States (US) and United Kingdom (UK) military personnel deployed in Iraq and Afghanistan have provided insights about these conditions. However only a few studies have assessed the mental health status of deployed troops longitudinally.

The UK and US armed forces are still deployed in Iraq and Afghanistan. A large cohort of military personnel from the UK deployed to Iraq and Afghanistan was evaluated in 2003 and 2009 [1], [2]. The sample included those surveyed in 2003 as well as troops who had been deployed subsequently [1]. In the follow up sample, the probable rate of post-traumatic stress disorder (4.0%) was similar to that in 2003. Prevalence of psychological distress too remained the same while alcohol misuse increased from 13.0% to 27% [1], [2]. Overall, the prevalence of mental disorders in the UK armed forces remained stable between 2003 and 2009.

These findings are in contrast to that from studies of US military personnel. Milliken et al found that US military personnel returning from Iraq reported higher rates of mental health problems six months after returning from deployment compared to immediately after their return. Among active duty personnel PTSD rates increased from 11.8% to 16.7%, depression from 4.7% to 10.3% and overall mental health risk from 17.0% to 27.1% [5]. Bliese et al found that reports of mental health problems were 2–5 fold higher at 120 days after returning from deployment than immediately after return [6]. Studies of Vietnam War veterans show that demobilization affects the mental health of military personnel because they have to adjust to civilian life. This contributed significantly to negative mental health consequences especially after the Vietnam War [7].

The contrasting findings highlight the need for further study. Many factors such as the type and duration of combat exposure, level of training, general support and availability of mental health services influence the prevalence of mental health problems. The 30-year civil war in Sri Lanka which ended in 2009 provided a unique opportunity to assess the mental health of military personnel from a non-Western nation. Sri Lankan Defense Forces had been engaged in combat operations during the past 30 years. During the period 2006–2009 the level of combat operations intensified resulting in high casualty rates [8], [9].

Since the end of combat operations in 2009, Sri Lanka Defense Forces have not engaged in combat. The role of the Sri Lanka Navy has changed from a combat force to one that is involved in patrolling territorial waters and contributing to developmental activities such as cleaning and maintaining of waterways. The main aim of this study was to assess the mental health status of the Navy Special Forces and regular forces three and a half years after the end of combat operations and compare it with the findings in 2009.

## Materials and Methods

### Study setting and participants

The study was carried out in the Sri Lanka Navy in February 2013, three and a half years after the conclusion of combat operations. The methodology was similar to that used in 2009 [10].

Study participants were selected using simple random sampling. The navy central database of personnel was used as the sampling frame. Participants were selected using computer generated random numbers. We did not explicitly try to identify personnel who had taken part in the 2009 survey although some may have participated in both surveys.

Participants were briefed regarding the nature and objectives of the study by personnel from the medical corps. The briefings were carried out in small groups at the place of deployment to improve response rates, aid clarification and facilitate provision of support for those with mental health problems. Participation was voluntary. The response rate was 91.7%.

### Inclusion and exclusion criteria

The inclusion and exclusion criteria were similar to that used in the 2009 study. Only personnel who had served continuously in combat areas during the one year period, May 2008 to 2009 were included in the study. Personnel who joined the units after the end of combat operations were excluded. Since there were no females in the Special Forces, females were excluded from the comparison group. A total of 220 Special Forces and 275 regular navy personnel were recruited to the study.

### Outcome measures

The 28 page questionnaire used in the study “Health of UK military personnel deployed to the 2003 Iraq war” was used as the data collection instrument [2]. Permission was obtained from the authors for the use of the questionnaire. The original questionnaire was translated into Sinhala and used in the 2009 study.

Mental health outcomes were measured using the following scales. Case definitions used were the same as those in the study of UK personnel deployed in Iraq [2]. Symptoms of psychological distress were identified using the General Health Questionnaire 12 (GHQ-12) a short screening questionnaire used in the general population and within community or non-psychiatric clinical settings. It is a 12 item questionnaire scored on a bimodal scale of 0-0-1-1. Cases were defined as individuals scoring 4 or more. PTSD was diagnosed using the 17 item National Center for PTSD checklist civilian version (PCL-C). This scale provides a diagnosis of PTSD according to the DSM-IV. Each item is scored on a scale of 1–5 and cases were defined as individuals scoring 50 or more. Fatigue was assessed using a 12 item version of the Chalder fatigue scale. Each item was coded as 0, 0, 1, 1 for categorical analysis and cases were defined as individuals scoring 4 or more. Hazardous alcohol use was identified using the WHO Alcohol Use Disorder Identification Test (AUDIT). The AUDIT has 10 items, each scored on a scale of 0–4. Cutoff of ≥8 identified hazardous and harmful drinking. Multiple physical symptoms were elicited using a checklist of 50 non-specific symptoms present in the past month. The same checklist was used in a previous study of UK military personnel [2]. Cases were defined as individuals with 10 or more symptoms. This case definition was the same used in the 2009 study.

Functional impairment was assessed using five questions from the SF-36 [11]. These questions explored the perceived functional impairment related to physical health or emotional problems. The areas explored were interference with normal social activities, problems with work or other regular daily activities as a result of physical health, cutting down on the amount of time spent on work or other activities, accomplishing less than would desired, limited in the kind of work or difficulty in performing work or other activities.

The General Health Questionnaire, Chalder Fatigue Scale, PTSD Check list and the AUDIT scale have been validated for Sri Lanka [12]–[15].

### Ethics statement

Ethical clearance was obtained from the Ethics Review Committee of the Faculty of Medicine, University of Colombo. Participation was voluntary and written informed consent was obtained from all participants. The questionnaire did not identify the participants by name.

### Statistical analyses

Analyses were done using SPSS version 17.0. Pearsons χ^2^ test was used to assess the difference between groups for categorical variables. Odds Ratios (OR) with 95% confidence limits (95% CI) were calculated to assess the differences in health outcomes between the two groups using multivariable logistic regression. Different models were built to control for confounders and identify risk factors. Model adequacy was tested using goodness of fit with the Hosmer Lemeshow test.

## Results

The sample consisted of 220 Special Forces and 275 regular forces personnel. The demographic characteristics of the sample are shown in Table 1. There were significant differences between the Special Forces and regular forces in age, marital status, educational status, rank, and usual duty. These differences were also present in the 2009 sample [10].

**Table 1:** Characteristics of the study sample.

	Special Forces n = 220 (%)	Regular Forces n = 275 (%)	Significance
**Age (years)**			
<25	39 (18.2)	24 (9.2)	χ^2^ = 35.9, df = 4 p<0.001
25–29	101 (47.2)	83 (31.9)	
30–34	51 (23.4)	79 (30.4)	
35–39	21 (9.8)	56 (21.5)	
40–49	2 (0.9)	18 (6.9)	
**Marital Status**			
Married	124 (56.6)	200 (73.0)	χ^2^ = 15.8 p<0.001
Single	95 (43.4)	73 (26.6)	
Previously married	0 (0.4)	1 (0.4)	
**Educational Status**			χ^2^ = 61.6 p<0.001
Less than GCE O’Level	103 (47.0)	54 (19.7)	
GCE O Level	97 (44.3)	129 (47.1)	
GCE A Level or higher	19 (8.7)	91 (33.2)	
**Rank (Current)**			χ^2^ = 9.1 p = 0.01
Commissioned officer	1 (0.5)	0 (0)	
Non-commissioned officer	19 (8.7)	57 (21.1)	
Other rank	199 (90.9)	213 (78.9)	
**Usual duty**			
Land combat	58 (26.6)	49 (18.4)	χ^2^ = 165 p<0.001
On board Naval vessels	125 (57.3)	28 (10.5)	
Others	35 (16.1)	189 (71.1)	

There were differences between the 2009 and 2013 samples. The 2013 sample was older (mean age 30.01 years; SD = 5.46) than the 2009 sample (mean age 27.63 years; SD = 5.02). Compared to 2009, in 2013 the personnel were more likely to be married (65.3% vs. 49.6%). In 2013, Special Forces were more likely to serve on board naval vessels (57.3%) than in land combat roles (26.3%). In the 2009 sample, 70.7% of Special Forces were engaged in land combat duties while 23.2% served on board naval vessels. Similar to the 2009 sample, in 2013 the majority of the regular forces were engaged in non-combat roles.

Compared to regular forces, a significantly higher number of Special Forces personnel had experienced potentially traumatic events (Table 2). The experiences reported in 2013 were similar to that in 2009. Commonest events experienced by the Special Forces in 2013 and 2009 were, seeing dead or wounded (86.8% vs. 81.5%), discharging weapons in direct combat (80.5% vs. 86.9%) and being involved in combat with enemy vessels (73.6% vs. 81.5%). Among regular forces the commonest experiences were seeing dead or wounded (65.1%) and handling dead bodies (44.0%).

**Table 2:** Potentially traumatic experiences during deployment.

	Special Forces n = 220 (%)	Regular Forces n = 275 (%)	Significance
Discharged weapon in direct combat	177 (80.5)	75 (27.3)	χ^2^ = 138.3, df = 1, p<0.001
Thought might be killed	118 (53.6)	99 (36.0)	χ^2^ = 15.4, df = 1 p<0.001
Involved in combat with enemy vessels	162 (73.6)	50 (18.2)	χ^2^ = 153.5, df = 1 p<0.001
Came under small arm fire	136 (61.8)	63 (22.9)	χ^2^ = 77.0, df = 1 p<0.001
Came under mortar, missile, artillery fire	112 (50.9)	63 (28.8)	χ^2^ = 41.9, df = 1 p<0.001
Experienced landmine strikes	7 (3.2)	11 (4.0)	χ^2^ = 0.23, df = 1 p = 0.63
Experienced hostility from civilians	4 (1.8)	7 (2.5)	χ^2^ = 0.30, df = 1 p = 0.59
Seeing dead or wounded	191 (86.8)	179 (65.1)	χ^2^ = 30.6, df = 1 p<0.001
Handled bodies	124 (56.4)	121 (44.0)	χ^2^ = 7.5, df = 1 p = 0.006
Aided wounded	93 (42.3)	101 (36.7)	χ^2^ = 1.6, df = 1 p = 0.21

Mental health outcomes were measured using several scales. Prevalence of psychological distress, fatigue, multiple somatic symptoms, PTSD and hazardous drinking were less in the Special Forces than in the regular forces while current smoking was more (Table 3). Unadjusted odds ratios show that these differences were significant for fatigue, multiple physical symptoms and hazardous drinking. When the model adjusted for demographic factors, the significant differences in fatigue and multiple physical symptoms disappeared but the significantly lower risk of hazardous drinking among Special Forces remained.

**Table 3:** Comparison of outcome between Special Forces and regular forces.

	Prevalence%Special Forces(95% CI)	Prevalence%RegularForces(95% CI)	UnadjustedOR(95% CI)N = 495	Adjusted OR*(95% CI)N = 495
Psychologicaldistress (GHQ-12)	6.5 (3.2–9.8)	10.2 (6.5–13.9)	0.61 (0.31–1.19)	1.37 (0.56–3.34)
Fatigue case	6.9 (3.5–10.3)	12.4 (8.4–16.5)	0.52 (0.28–0.99)	0.80 (0.34–1.89)
Multiplephysicalsymptoms	4.1 (1.5–6.8)	10.5 (6.7–14.13)	0.37 (0.17–0.80)	0.39 (0.14–1.04)
Hazardousdrinking(AUDIT≥8)	17.8 (11.8–21.8)	25.1 (19.9–30.3)	0.60 (0.40–0.98)	0.48 (0.26–0.88)
Currentsmoking	54.8 (47.9–61.2)	51.3 (45.3–57.2)	1.15 (0.81–1.64)	0.86 (0.52–1.40)

*Adjusted for age, marital status, education, rank and usual duty.

Table 4 shows the association between mental health problems and functional impairment. Psychological distress, fatigue and multiple somatic symptoms were associated with all measures of functional impairment. Hazardous drinking (AUDIT Score ≥8) was associated with interference in social life, limitation in work and difficulty in performing work.

**Table 4:** Association between mental health problems and functional impairment.

	Totalsamplen (%)	*AUDIT≥8n = 107 OR	*Psychologicaldistress (GHQ12)	*Fatigue	*Multiplesomaticsymptoms
Health interferedwith social life	157 (23.40)	OR 2.13 (1.28–3.55)	OR 9.90 (4.72–20.75)	OR 5.11 (2.64–9.91)	OR 7.22 (3.41–15.31)
Cut down time onwork/otheractivities	82 (12.22)	OR 1.08 (0.55–2.11)	OR 8.42 (3.70–19.16)	OR 4.27 (1.96–9.27)	6.82 (3.04–15.32)
Accomplishedless thanwouldlike	89 (13.26)	OR 1.02 (0.48–2.18)	OR 4.80 (2.08–11.10)	OR 4.96 (2.27–10.87)	OR 5.39 (2.24–12.97)
Limited in typeof work	101 (15.05)	OR 2.09 (1.12–3.89)	OR 5.60 (2.65–11.80)	OR 8.45 (3.94–18.15)	OR 4.66 (2.04–10.64)
Difficultyperformingwork	165 (24.59)	OR 1.82 (1.06–3.13)	OR 5.60 (2.65–11.80)	OR 6.94 (3.45–13.98)	OR 4.72 (2.22–10.02)

*Adjusted for age, marital status, education, rank, service type and role within unit.

Table 5 compares the prevalence of mental health outcomes in the 2009 and 2013 samples. Compared to 2009 in 2013, in the Special Forces, prevalence of psychological distress (6.2 vs. 6.5) and fatigue (5.4 vs.6.9) showed marginal increase while hazardous drinking (17.4 vs. 16.8) and multiple physical symptoms (5.8 vs. 4.1) showed a marginal decrease. In the regular forces, prevalence of psychological distress, fatigue and multiple somatic symptoms declined and hazardous drinking increased from 16.5% to 25.7%. The prevalence of smoking increased significantly in both Special Forces and regular forces during this period. The prevalence of PTSD had declined from 1.9 in the Special Forces to 0.9 (−0.4–2.2) and in the regular forces from 2.07 to 1.1(−0.1–2.3).

**Table 5:** Comparison of prevalence of mental health problems 2009 and 2013.

	2009 SpecialForces(95% CI)	2013 SpecialForces(95% CI)	2009RegularForces (95%CI)	2013RegularForces(95% CI)
**Psychological** **distress (GHQ-12)**	6.2 (3.2–9.1)	6.5 (3.2–9.8)	15.3 (11.8–18.8)	10.2 (6.5–13.9)
**Fatigue case**	5.4 (2.6–8.2)	6.9 (3.5–10.3)	18.5 (14.7–22.2)	12.4 (8.4–16.5)
**Multiple physical** **symptoms**	5.8 (2.9–8.7)	4.1 (1.5–6.8)	13.4 (10.1–16.7)	10.5 (6.7–14.1)
**Hazardous drinking** **(AUDIT≥8)**	17.4 (12.7–22.0)	17.8 (11.8–21.8)	16.5 (12.9–20.1)	25.7 (19.9–30.3)
**Current smoker**	23.6 (18.8–28.8)	54.8 (47.9–61.2)	14.3 (10.9–17.7)	51.3 (45.3–57.2)
**Post traumatic** **stress disorder** **(PTSD)**	1.9 (0.2–3.6)	0.9 (−0.4–2.2)	2.7 (1.1–4.2)	1.1 (-−0.1–2.3)

## Discussion

This is one of the few studies to explore the long-term mental health impact of deployment in combat areas. The study carried out in the Sri Lanka Navy found that during the three and a half year period after combat operations ended, mental health problems declined among regular forces, while there was no substantial change among Special Forces. However in the regular forces hazardous drinking increased and in both Special Forces and regular forces smoking rates almost doubled [16], [17].

In 2009 we reported that the prevalence of mental health problems was less in the Special Forces compared to regular forces although Special Forces experienced more potentially traumatic events [10], [18]. After three and a half years, the findings were similar although the difference between the groups was not statistically significant. In the three and a half year period between 2009–2013 the mental health outcomes improved more in the regular forces than in the Special Forces.

A large cohort study of UK troops deployed in Iraq and Afghanistan found that there was no significant change in mental health problems in the follow up sample in 2009 compared to 2003 [1], [2]. In US military personnel, several studies have reported that rates of mental health problems have increased since returning from deployment [3]–[6]. UK and US troops continued to be deployed in high risk areas in Afghanistan unlike Sri Lanka Navy personnel who have not been engaged in combat since 2009 [1], [2]. This could be one reason for the reduction in mental health problems among regular forces of the SLN unlike in US and UK troops.

Deployed reservists in the UK and US National Guard and Army Reserve soldiers report substantially higher rates of interpersonal conflicts, PTSD, depression and overall mental health risk [3]–[5]. National Guard and Army Reserve soldiers return to civilian status after deployment and have to thus make major adjustments and also miss the support of comrades [5]. In Sri Lanka the military personnel engaged in combat operations were mainly regular forces, not reservists. Although there was a prolonged civil war, there was no conscription. SLN personnel were not demobilized after cessation of combat operations and therefore continued to receive support from colleagues, access to health services and job security. All these factors would have contributed to the improvement of mental health status among regular forces.

In the period immediately after end of combat operations we found that the prevalence of PTSD was low among both Special Forces (1.9%) and regular forces (2.7%) [10]. These rates decline even further in the three and a half years since the end of combat operation. Studies of US military personnel returning from Iraq indicate that in the short term (up to 6 months) there is an increase in the reported prevalence of PTSD and other mental health problems [5], [6]. In a long-term cohort study of UK personnel, PTSD rates increased from 3% after one year to 5.8% after four years [1].

Psychological distress increased marginally (6.2% to 6.5%) in the Special Forces and declined (15.3% to 10.2%) in the regular forces. The General Health Questionnaire is a good general measure of mental health, and in our sample caseness was strongly associated with functional impairment.

A cause for concern was the increase in hazardous drinking in the regular forces and the substantial increase in rates of smoking in both the Special Forces and regular forces. In the Special Forces prevalence of smoking increased from 23.6% to 54.8% and in the regular forces from 14.3% to 51.3%. In the 2009 study the low rates of smoking were attributed to smoke free policies in the military installations and a declining trend in smoking among the general population [17]. Surveys of the general population show a decline in the prevalence of smoking in urban areas from 29.9% in 2009 to 17.2% in 2011 and in rural areas from 24.4% to 18.5% [19]–[21]. The relaxation of smoke free policies and the easy availability of cigarettes due to increased accessibility in former combat areas may account for the increased rates of smoking. Hazardous drinking increased among regular forces but not the Special Forces. Limited availability of alcohol was thought to be one reason for the low rates of alcohol problems in 2009 [16]. The end of the war would have eased restrictions on access to alcohol. The availability of alcohol in the Northern and Eastern provinces of the country where these troops are deployed also would have encouraged higher rates of alcohol consumption. In 2013 majority of the sample consisted of non commissioned officers (NCO) who have more access to alcohol in the camp than lower ranks.

Although the follow up study did not reveal dramatic increase in mental health problems it is anticipated that the Navy personnel will have to adjust to different roles and responsibilities. Since the end of combat operations they interact more with civilians, engage in development work and do not have a combat role at all. This could lead to different types of issues such as interpersonal conflicts and role conflicts. Although PTSD rates were low, personnel with mental health problems reported significant functional impairment. The military will have to take into account the change in the role of personnel and the need to provide mental health care or those affected. They also need to focus on prevention of tobacco and alcohol use among personnel.

This study had several limitations. We did not follow up the same sample that was surveyed in 2009. However some personnel may have participated in both studies. There were differences in demographic factors between the two samples which could have affected the outcome. The mean age of the 2013 sample was about 3 years more than the 2009 sample. This is to be expected as we did not include new recruits in the sample and those who were already in service have aged. The 2013 sample also included more married personnel. The end of combat operations and less strenuous working conditions may have encouraged personnel to get married. However these differences alone do not explain the changes seen during the four years.

### Conclusions

In Sri Lanka Navy personnel, three and a half years after end of combat operations, mental health problems declined among regular forces while there was no significant change among Special Forces. Hazardous drinking among regular forces and smoking in both Special Forces and regular forces have increased. Appropriate adjustment to peace time roles and continued support from the Navy may have contributed to the improvement in mental health.
